# Parent Support Programmes for Families Who are Immigrants: A Scoping Review

**DOI:** 10.1007/s10903-021-01181-z

**Published:** 2021-03-26

**Authors:** Lotta Hamari, Jenni Konttila, Marko Merikukka, Anna-Maria Tuomikoski, Petra Kouvonen, Marjo Kurki

**Affiliations:** 1 Nursing Research Foundation, Asemamiehenkatu 2, 00520 Helsinki, Finland; 2 Department of Nursing Science, University of Turku, Turku, Finland; 3 Department of Nursing Science and Health Management, University of Oulu, Oulu, Finland; 4 Itla Children’s Foundation, Helsinki, Finland; 5 Finnish Institute for Health and Welfare, Helsinki, Finland; 6 Oulu University of Applied Sciences, Oulu, Finland; 7 Finnish Centre for Evidence-Based Health Care: A JBI Centre of Excellence, Helsinki, Finland; 8 Department of Child Psychiatry, University of Turku, Turku, Finland

**Keywords:** Parent support programmes, Emigrants and immigrants, Refugees, Scoping review

## Abstract

Parental support is of paramount importance in the promotion of positive parenting, strengthening parenthood and protecting children from disadvantages due to immigration experiences. The aim was to describe what is known about parent support programmes targeted to families who are immigrants. Electronic databases and the grey literature were systematically and comprehensively searched with no time/language restrictions. JBI approach and PRISMA-ScR were used to guide the review. N = 88 articles were sourced. Interventions were targeted to improve parental practices, skills and family wellbeing, usually through group-based methods. Most interventions included components of positive parenting and family communication. Identifying the needs of the target group and cultural tailoring were reported to be highly important in gaining acceptability, promoting engagement and producing benefits. Parent support programmes for families who are immigrants potentially improve positive parental practices and families’ wellbeing. There are many applicable and effective interventions to be exploited.

**Supplementary Information:**

The online version contains supplementary material available at 10.1007/s10903-021-01181-z.

## Introduction

Currently, more than 70 million people worldwide have been forcibly displaced from their homes. Over 25 million of these forcibly displaced people are refugees and the majority of them are children under the age of 18 [1]. Moving to another country is a stressful process and a major life change for anyone but is something that can negatively affect children in particular [2–5]. Severe stress is harmful during early childhood when the developing brain is very sensitive to environmental influences [3]. During the ‘refugee experience’ parental presence and support are of paramount importance in protecting children from negative physical and psychosocial symptoms [3, 6]. In reality, caring and protective parenting can be threatened due to a variety of negative influences in terms of the resettlement process and pre-migration experiences [7]. This review is not limited to including only studies with refugees, since we focus on all forcibly displaced people. Although these populations face similar acculturation challenges, the migration process of refugees might include more traumatic experiences. However, all forcibly displaced might be in e need to receive parent support, and therefore all forcibly displaced are in the focus of the current review.

Migration and the resettlement process are both associated with many stressors. These stressors, include the psychological strain or distress of the migratory experience and the acculturation process, with consequences for mental health. For example, it has been estimated that the prevalence of post-traumatic stress disorder (PTSD) among migrants (i.e. refugees and labor migrants) is 47% [5, 8] and in war-affected children who are refugees 30.4% [9]. The prevalence percentages do however vary greatly across studies being 19.0–52.7% for PTSD, 10.3–32.8% for depression, 8.7–31.6% for anxiety disorders and 19.8–35.0% for emotional and behavioural problems [10]. Nevertheless, it is clear that these multiple stressors and the possibly that the less responsive caregiving of offspring, increases the risk of developing health problems and psychopathology in children who are refugees [3, 9–14]. Family-based approaches, such as parent support programmes are suggested as a way to improve children's emotional and behavioural problems [15]. As such, there is a clear and urgent need for better evidence-supported information about parenting support methods for immigrant families to ensure health equality and access to support [6].

Parent support programs are defined as programs that are aiming to strengthen and support parenting abilities and promote new competencies so that parents have the skills and knowledge needed to conduct child-rearing practices [16, 17]. Parent support programs are also aiming to enable parental competencies that promotes providing their children experiences and opportunities which promote child learning and development [16]. In this review, by parent support programs we also mean those methods and programs that are targeted to parents and which are aiming to improve parent’s and children’s health and wellbeing and to prevent maltreatment and abuse of their children. The included studies must contain a family/parent component meaning that the intervention must be targeted solely or partly to parent/s who are immigrants.

Preliminary searches conducted in October 2019 revealed that little is known about parent support programmes targeted at immigrant and refugee populations. In 2017, The Lancet Psychiatry published a call for parenting interventions for mothers who are refugees to promote the healthy development of their children younger than 3 years [6]. This petition also certifies the need for evidence in parenting support programmes for immigrant families. As our initial search for reviews on this topic support the thesis that the evidence base for parent support programmes for immigrant families seems to be fragile [4, 6], we decided to conduct a scoping review in order to gather and summarise existing knowledge in this field and to provide a broad view of what is known about the topic to date.

The aim of this scoping review is to describe what is known about parent support programmes targeted at families who are immigrants. The results of this review will help in targeting, developing, utilising and implementing new methods in child and family services to support parents who are immigrants. The main review question is answered in relation to the next four clarifying questions: (1) to what purpose are the parent support programmes targeted, (2) how were the participants reached, (3) what components do these parent support programmes include, and (4) what outcomes and results have the parenting support programmes demonstrated (if any).

## Methods

**Fig. 1:**
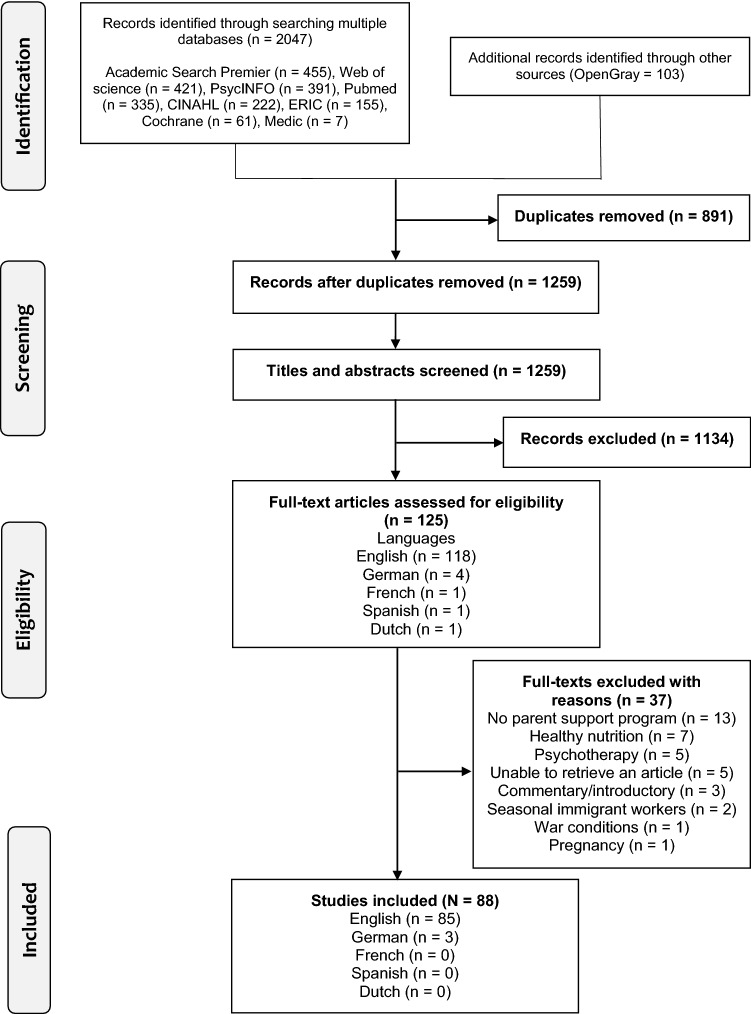
The PRISMA flow diagram

This scoping review was conducted according to the guidance provided by JBI [18] including the application of a PRISMA-ScR statement [19]. The review questions were formulated based on the applicable parts of the template for intervention description and replication (TIDieR) checklist [20]. The search strategy used and the study selection process are illustrated in the Flow chart (Fig. 1). The protocol was registered prior to conducing the literature searches in Open Science Framework (OSF 2019-11-01). Since this is a review article, there was no need for ethical approval from the institutional and/or national ethical review committee.

### Search Strategy and Data Sources

The databases Academic Search Premier (EBSCO), Web of Science, PsycINFO (EBSCO), PubMed/Medline, CINAHL, ERIC (EBSCO), Cochrane Library, and Medic (Finnish database) were searched for relevant literature (11/2019). OpenGray was searched for the relevant ‘grey’ literature. Search terms were identified through an initial search and all identified keywords and index terms were utilised across all included databases. The search terms included such as emigrants, immigrants, emigration, immigration, refugees, asylum seeker, displaced person, displaced people, parenting, parents, caregivers, parent–child relations, family, mother, father, guardian, parent support program, parenting support program, parent support, parenting support, parent program, parenting program, parent training, parenting training, parent intervention, parenting intervention, parent education, parenting education, family support, family program, family training, family intervention, family education, social support, health promotion, counselling, support, program, training, intervention as Main Heading (Descriptors) and free word queries. The full search strategy and queries are presented in Online Appendix 1. The search was conducted together with an information specialist.

### Study Selection

The study selection was at all stages of selection performed based on the inclusion criteria. First, the titles and abstracts were screened and thereafter the full texts of the selected articles. The selection process was conducted by two independent reviewers. Disagreements between the two reviewers were solved by discussion and consensus or by the decision of a third reviewer [18].

Inclusion and exclusion criteria were developed using the PCC- model where P is for population, C for concept and C for context [18]. In this review, the *population* of interest is first generation immigrant parent(s) with a child/children under 18 years of age. The key *concept* of this review is parent support programmes. Parent support programmes are defined as programmes that aim to strengthen and support parenting abilities and promote new competencies so that parents have the skills and knowledge needed to conduct child-rearing practices. Parent support programmes also aim to enable parental competencies that promote the provision of their children with experiences and opportunities which promote child learning and development [16]. The included studies must contain a family/parent component meaning that the intervention must be targeted solely or partly to parent(s) who are immigrants. In this review, the *context* of interest is the new host country including health and social care settings, school, kindergarten, community, jail, refugee centres, detention centre, immigration detentions, refugee camps, home and remote/digital environments. The new host country should be classified as an upper-middle-income economy or high-income economy (HIC) based on the World Bank criteria [21]. The inclusion and exclusion criteria are presented in Table 1.

**Table 1:** Inclusion and exclusion criteria

	Inclusion criteria	Exclusion criteria
Population	First generation immigrant parent/parents with under 18-year-old child/children (documented and undocumented residents, refugee claimants, refugees, asylum seekers in this definition of immigrant are included)	Economic migrants, students, and skilled workers or temporary foreign workers (also temporary farm/harvest workers)
Concept	Parent support program/programs which include a component targeted solely or partly to parents	If the parent support program were targeted to medical specialities listed below they were excuded: Pregnancy, safe birth, childbirth Oral health Healthy nutrition and vitamin intake or obesity prevention Immunization Sexual health Family planning/birth control/fertility/genetic counseling Family therapy or psychotherapy Tuberculous infection therapy Pesticide safety Malaria treatment/tropical medicine
Context	Any context in the new host country	Studies that has been conducted at war conditions
Publication type	Scientific publications that have gone through a peer-review process and official reports	Commentaries, introductory journal articles, editorials and letters to the editor are excluded
Study design	Qualitative, quantitative and mixed-method research studies and reviews including feasibility and implementation studies	

A total of 2047 original reports and articles were identified through the selected databases and 103 additional records were identified through OpenGray. After removing duplicates, 1259 records were screened by title and abstract and 125 full-texts eventually screened. A total of 88 reports and articles were included in the final analysis.

### Data Charting

The JBI approach and PRISMA-ScR were used to guide data charting and the reporting of the results. A data charting table (Online Appendix 2) was made and tested before its use by the researchers. Information was collected on the author(s), year of publication, publication/study country, aim of the study or report, study methods, study population and key findings (if applicable). The data charting table was collated by two researchers .

### Data Analysis/Synthesis of Results

Data was analysed using the narrative synthesis method, an approach often utilised when the included studies are heterogeneous in terms of methods, participants or data [18, 22]. Frequencies were computed to describe particular details.

The narrative synthesis of evidence was undertaken by grouping the studies by their methodology, interventions (parent support programmes) and participants. After that, we further summarised the interventions based on the research questions and summarised their purposes, recruitment strategies, procedures, delivery methods and tailoring. In addition, the benefits of the parent support programmes’ (research question 4) were summarised based on the selected studies’ methodology. NVivo 11 (QSR International 2020) was used in data handling and analysis.

## Results

### Characteristics of the Selected Studies

**Fig. 2:**
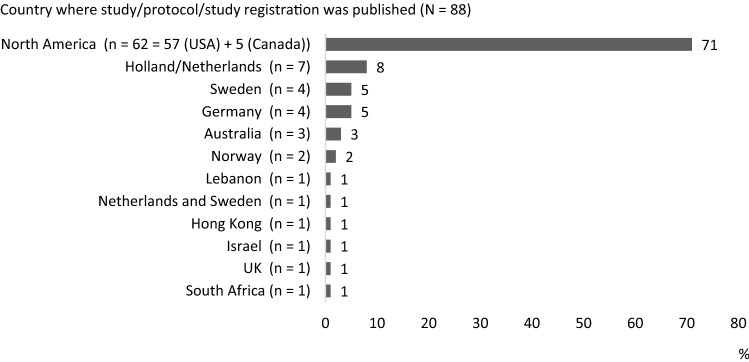
Countries where the studies were conducted

A total of N = 88 articles were included in this review. The majority of the studies were conducted in North America. The countries of origin are reported in Fig. 2. In two studies, the first author affiliation was in USA but the intervention was conducted in another country (Thailand and Turkey) [23, 24].

**Fig. 3:**
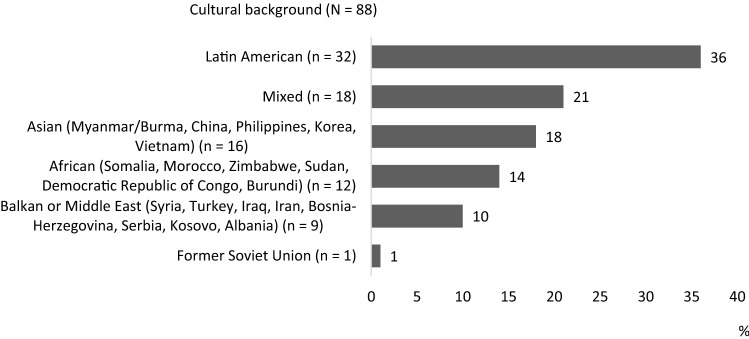
Cultural backgrounds of those targeted by the programmes

Most of the parent support programmes were targeted at parents with a Latin American cultural background (n = 32) [25–53]. The cultural background of the participants or target participants in parent support programmes is reported in Fig. 3. In a total of 18 studies, the study participants were from mixed cultural backgrounds or the cultural background was not specified [4, 54–70] (Fig. 3).

**Fig. 4:**
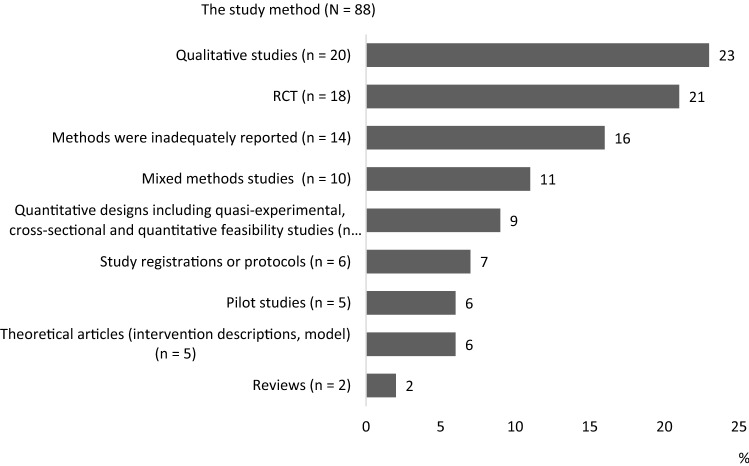
Methods of the included studies

The study methods of the included studies are reported in Fig. 4. Most of the studies were qualitative (n = 20) [26, 31, 39, 42, 49, 57, 68, 70–82] and RCT studies (n = 18) [23, 34, 36, 38, 44, 58, 64, 83–93]. A total of 14 of the studies lacked a clear methods section or the methods were inadequately reported [27, 28, 30, 32, 33, 45, 55, 56, 59, 66, 94–97] (Fig. 4).

### Recruitment of the Participants

Participants were recruited into parent support programmes through community-serving agencies/organisations [25, 26, 33, 57, 72, 80, 84, 96, 98–101] and local communities [23, 26, 31, 35, 36, 44, 50, 68, 71, 73, 74, 80, 82–84, 88, 89, 92, 94, 96, 102–105]. Research and parent support programme information was shared in the community for example by distributing flyers [39, 56, 98–100, 104–107] or via direct invitation (in-person contact or personal contact by phone) by local community leaders, health workers, religious leaders, interpreters, research coordinators, researchers, school counsellors or teachers [32, 39, 50, 71, 80, 84, 98]. In addition, studies also recruited participants from public services e.g. kindergarten [55, 64, 70, 76], schools [29, 30, 32, 33, 37, 38, 40, 56, 58, 72, 80, 84–87, 90, 94, 95, 104], language schools [73, 74, 107, 108], churches or religious organisations [80, 94, 105, 109, 110], social services, or through social worker [29, 38, 84–87, 104], child welfare services [84], health care clinics [36, 44, 50, 54, 55], mental health services or through mental health worker [44, 49, 50, 58, 71, 85, 104, 105], initial registration centre [78] and child protective services [85, 95, 104]. Some studies reported using social media [98], television [28], radio [100, 107] and newspaper advertisements/invitations [28, 100, 106]. A word-of-mouth strategy was also much used to recruit participants [28, 36, 44, 62, 80, 84, 89, 101, 105–107].

Personal invitations were seen as effective strategies in recruitment, including word-of-mouth and direct invitations by community leaders or service providers [80, 84]. Bilingual communication was seen as important [84]. Community members were also seen as important links between the parent support programme providers and the target participants. In many studies, community members were engaged in several roles and at different phases of the programme and worked as interventionists, research assistants, and/or as community advisory board members [92].

### Identified Interventions to Support Immigrant Parents

A total of 14 interventions were studied two times or more in original studies or reports and two study protocols were designed to use the same intervention. These 14 interventions were Padres Informados/Jóvenes Preparados (PI/JP) [25, 31, 98], Happy Families Program (HFP) (adapted from the Strengthening Families Program) [23, 88], Generation PMTO (Parent Management Training- Oregon model) [43–45, 83, 84], Supported Playgroups [57, 68], Caregiver Supported Intervention (CSI) [102, 103] and incredible years [58, 73, 74, 85, 95], CAPAS-Original/CAPAS-Enhanced (based on PMTO) [36, 45, 105], Family Communication [38, 39], Connect- Programme (Ladnaan- programme = Connect- programme + information on Swedish society) [75, 86, 87], Familias Unidas [40, 41], Migrant Education Event Start (MEES) [47, 48], Social Support Intervention [81, 100], Fortalezas Familieares (FF): Family Strengths [49–51] and Coffee and Family Education and Support (CAFES) [93, 101]. These interventions are presented (rationale of intervention, methods to reach participants, intervention procedures and materials, delivery of intervention and tailoring) in Table 2, and synthesised in the following sections. A total of 33 interventions appeared just once and the names and rationales of these interventions are presented in Table 3.

**Table 2:** Parent support programmes that were studied more than once

	Name of intervention	Rationale of intervention	Intervention procedures/materials	Delivery of intervention	Tailoring
Allen et al. [25], Garcia-Huidobro et al. [31, 98]	PI/JP: Padres Informados/Jóvenes Preparados	To prevent Latinx youth substance use by improving parenting practices, parent–youth interpersonal skills, and youth social competencies	Group sessions; one-to-one (online) component for those who did not attend to group sessions; parent training, parent/youth relational skill building, youth training; conversations, exercises and roleplay	Eight 3 h sessions (incl. 30 min for dinner): 4 sessions for parents, 1 session for parents and youth in independent topics, 3 sessions for parents and youth in parallel topics; by bicultural and bilingual facilitators with appropriate education and working experience; in Latinx community serving agencies	Cultural adaption via CBPR approach
Annan et al. [23], Puffer et al. [88]	HFP: Happy Families Program (adapted from the strenghtening Families Program)	To support parenting skills to prevent mental health problems	Parallel group sessions for caregivers and children; lectures, demonstrations, roleplay, in vivo practice	12 (14) weekly 2.5 h sessions (incl. lunch together); by trained facilitators; in local community services	Cultural adaptation via qualitative approach
Ballard et al. [71], Bjørknes and Manger [84], Bjørknes et al. [83], Parra Cardona [43], Parra Cardona et al. [44]	GenerationPMTO: parent Management Training- Oregon model	To help parents manage children’s misbehavior	Group sessions; encouragement of positive behavior, positive involvement; monitoring, problem-solving, homework exercises	10–18 weekly 1.5 (2) hours sessions; by trained providers (simultaneous translation)	Cultural adaptation via qualitative approach
Guo and Gray [57], Warr et al. [68]	Supported Playgroups	To support families from culturally and linguistically diverse backgrounds	Cross-cultural playgroups, language specific playgroups, playgroups for refugees and asylum seekers; organized social and play-based activities	Playgroup-sessions; by playgroup facilitators; in community-based organizations	Need-based tailoring
ISRCTN33665023 [103], ISRCTN22321773 [102]	CSI: Caregiver Supported Intervention	To strengthen caregiver’s psychosocial wellbeing and parenting	Group sessions; interventions to reduce stress, strengthen psychosocial wellbeing, and strengthen parenting by increasing warm and responsive parent–child interactions and decreasing harsh parenting	Nine 2 h sessions; by trained and supervised non-mental health specialist; in local community-based organization	Gender-based tailoring
Kim et al. [73, 74], Lau et al. [85, 95], Leijten et al. [58]	Incredible years	To enhance parental skills	Group sessions; teaching parents parent–child play, praise and rewards, coaching of social, emotional and academic skills, effective limit setting, and handling misbehavior (e.g., ignore and time-out techniques); lectures, videotaping, home works, experience sharing	12–18 weekly 2 h sessions; by group leaders; in participants’ children’s school	Cultural tailoring
López-Zerón et al. [36], Parra Cardona et al. [45, 53]	CAPAS-Original CAPAS-Enhanced (based on PMTO)	To teach parenting strategies to reduce coercion and increase positive parenting practices	Parent group sessions; detailed session agendas, objectives, exercises, role-plays and group process suggestions adapted from PMTO	12 weekly 1.5 h sessions (incl. dinner for whole family); by two facilitators; in local religious organization	Cultural tailoring
McNaughton et al. [38, 39]	Family Communication	To reduce incendiary communication	Small group-format; 4 groups for mother–child dyads; discussions, group activities (for mother–child dyads together and separately); roleplay, communication skills practices, home works, PowerPoint presentations	Six 2 h sessions; by project coordinator, in a classroom	Cultural tailoring; Linguistical tailoring
Osman et al. [75, 86, 87]	Connect- Programme (Ladnaan- programme = Connect- programme + information on Swedish society)	To strengthen the parent–child relationship; to give information about Swedish social and healthcare system	Group sessions; culturally tailored social information, parental reflection; group lectures, workshops, discussions; childcare services during sessions	12 weekly 1 to 2 h sessions (incl. beverages and snacks); by group leaders; in participants neighborhood	Cultural tailoring
Pantin et al. [40, 41]	Familias Unidas	To prevent drug abuse and antisocial behavior	Group sessions; three stages program: 1. set specific objectives, 2. promotion of parental investment, 3. fostering parenting skills; group discussions, problem posing, participatory exercises	Weekly 1 h family centered multiparent groups during 9 months; by female facilitators	Not mentioned
St. Clair and Jackson [47], St. Clair et al. [48]	MEES: migrant education event start	To enhance parental involvement to children’s education; to support children’s learning at home	Group sessions; modeling with an opportunity for supportive practice; educational and networking sessions	25 one-hour training sessions over the course of the school year; by parent educators	The content of the parenting curriculum was adapted from child’s kindergarten curriculum
Stewart et al. [81, 100]	Social support intervention	To offer social support for refugee parents of young children	Group sessions guided by challenges and parent preferences; online videos, discussions, reflective listening, problem solving; follow-up support through individual meetings	8 face-to-face support groups bi-weekly over 7 months; by a like-ethnic and like-gender peer mentors	Cultural tailoring
Valdez and Martinez [49], Valdez et al. [50, 51]	FF: fortalezas familieares; family strengths	To support the mother with depression and the family system	Multi-family group format; interpersonal and group process; identification of feelings and stressful situations, developmentally appropriate activities	12 (14)- week intervention including separate intervention groups for adults and youth (incl. culturally-representative meal); by clinical facilitators; in the evenings centrally-located community agency	Roughly age matched groups for youth; cultural tailoring
Weine et al. [93, 101]	CAFES: Coffee and Family Education and Support	To emphasize family strengths and resilience	Group sessions in 4 different phases; discussions from topics concerning the issues of mental health problems and mental health services	9 multiple- family group sessions over 16 weeks including 15-min didactic talk followed by 1-h family discussion; by three female group facilitators	Cultural tailoring

**Table 3:** Name and rationale of the parent support programmes that were studied once

	Name of intervention	Rationale of intervention
Azziz-Baumgartner and Wilson [26]	FF: Familias Fuertes	To prevent teens’ substance abuse and other behavior problems, and to enhance parenting and family strengths
Betancourt et al. [92]	FSI-R: Family Strengthening Interventions for Refugees	To promote youth mental health and family relationships
Cowell et al. [29]	IMFBH: Interaction Model of Family Health Behavior	To promote mental health of families with Mexican background
Cwikel et al. [54]	M2M: Mom to Mom	To help women cope with the first year of parenting through home visits of volunteer mothers
Dababnah et al. [72]	PTCs: Parent-Teacher Cooperatives	To train and support teachers to work with refugee children with autism
Dumka et al. [30]	Puentes	To prevent school disengagement and mental health problems in Mexican origin 7th graders
Gonzalez et al. [32]	HSD Model	To involve minority parents to their children's school
Hendrickson et al. [34]	Promotora	To increase maternal self-efficacy and child safety
Knox et al. [35]	FAST: Families and Schools together	To prevent/reduce aggression among elementary-school aged children of immigrant Latinx parents
McNaughton et al. [37]	MAPS: Mexican–American Problem Solving Program	To assist Mexican immigrant mothers and their children develop problem-solving strategies in dealing with stressors
Nagoshi et al. [99]	FPNG: Families: Preparing the New Generation	To develop parenting skills and to enhance parents’ knowledge about adolescent development
NCT02829086 [60]	Family Stress and Conflict Management	x
NCT03040154 [124]	Video Documentary: For Our Children's Future	x
Nieuwboer and van't Rood [61]	IDEAL: Integrating Disadvantaged Ethnicities through Adult Learning	To support non-western migrant mothers without previous formal education in their efforts toward achieving social integration in a Western host society
Paris [42]	Visiting Moms	To serve newly arrived mothers and children
Pejic et al. [97]	PAIF: Preventive and Access Interventions for Families	To provide preventive services in a community-based setting
Ponguta et al. [62]	MOCEP: Mother–Child Education Program	To improve parental practices and skills to promote holistic early child development (in children 3-to-11 years of age), with a focus on school readiness
Renzaho & Vignjevic [112]	African Migrant Parenting Program	To improve parenting practices
Rivera [46]	Community Learning Centers (Centros Comunitarios de Aprendizaje, or CCA)	To allow Hispanic parents to learn marketable computer skills
Samarasinghe [63]	SAFRI: Samarasinghe Refugee Family Intervention Model	To manage the acculturation process, to integrate the family into society and to achieve stable family relationship
Schnur et al. [77]	JCCA: Jewish Child Care Association	To serve low-income immigrant families from the former Soviet Union
Schulz et al. [64]	Triple P	To prevent child behavioral disorders for families with children aged 3 to 6 years
Singh et al. [79]	IL: Book Distribution Program	To effect a change at the immediate level of the microsystem (i.e., the child’s home life)
Sritharan and Koola [65]	SAAAC: South Asian Autism Awareness Center	x
Umubyeyi and Harris [110]	Training in non- violent alternatives	To teach parents to bring up their children using non-violent methods and children can learn to interact non-violently with others
van Es et al. [67]	FAME: Family Empowerment	To support families in asylum centers and family facilities
Weine et al. [109]	TAFES: Tea and Family Education and Support	To help families to cope together under the stresses of survival and displacement and to improve the families’ ability to obtain appropriate care for possible mental health consequences of torture from sources outside of the family
Williamson et al. [89]	Madres a Madres (Mother to mother)	To support motherhood of Latinx mothers
Wong et al. [90]	Bridges	x
Yagmur et al. [91]	VIPP-SD: Video-feedback Intervention to promote Positive Parenting (VIPP-TM for Turkish Minorities)	To support parents to show more sensitive responsiveness to child’s signals
Ying [108]	SITICAF: Strengthening of Intergenerational/Intercultural Ties in Immigrant Chines American Families	To bridge the intergenerational and intercultural gap in Chinese American immigrant families
Ying [107]	SITIF: Strengthening of Intergenerational/Intercultural Ties in Immigrant Families	To strengthen the intergenerational relationship between immigrant parents and their school age children and adolescents
Yuen [70]	GPEP: Group Parent Education Program	To develop parenting knowledge and skills among new immigrant parents

### Rationales of Interventions

The parent support programmes were generally targeted either to support [23, 49–51, 57, 68, 81, 88, 93, 100, 101], enhance [58, 73, 74, 85, 95] or to strengthen [36, 44, 45, 105] parental skills and parenthood [102, 103] or to prevent youth [25, 31, 40, 41] and children’s [43–45, 71, 83, 84] behavioural problems. Interventions were also targeted at enhancing family communication [38, 39], strengthening the parent–child relationship [75, 86, 87] and enhancing parental involvement in children’s education [47, 48]. Notably, the Connect-programme [75, 86, 87] was extended to the Ladnaan-programme which aimed also to provide information about the Swedish social- and healthcare system along with the Connect-programme. It was also seen as important that refugee parents got to know the policies and practices of their new host country. Parental support programmes also had various indirect aims and, for example, PI/JP intervention [25, 31, 98] aimed to prevent substance use among youths by improving parental skills.

### Components of the Interventions

Interventions were mainly well designed and structured. Methods used in the delivery of the interventions were versatile, including demonstrations [23, 88] and problem-solving [43–45, 71, 83, 84], online- and video-material [58, 73, 74, 85, 95], conversations [25, 31, 98] and discussions [38, 39, 75, 86, 87], exercises in the group sessions [25, 31, 98], homework [38, 39, 43–45, 58, 71, 73, 74, 83–85, 95], roleplaying [23, 25, 31, 88, 98] and social activities [57, 68]. The purposes of the tasks and materials were, for example, to strengthen positive parenthood and build relational and communicational skills with children [58, 73, 74, 85, 95], encourage positive behaviour [43–45, 71, 83, 84], and offer emotional and social support [54, 89]. Materials were tailored to be suitable for each target population. Online-materials were offered if participants were hindered in terms of physically participating in the actual parent support programme sessions [25, 31, 98].

Most of the interventions (FF, PI/JP, HFP, PMTO, incredible years, CAPAS, family communication, connect-programme, social support intervention) included, on average, 6 to 18 weekly sessions while the duration of these sessions varied between 1 and 2.5 h. In some interventions (PI/PJ, HFP, CAPAS, Connect-Programme), sessions included lunch, dinner or a snack and beverages, since it was seen as important that parents and children had the possibility to eat together [23, 44, 45, 88, 105]. Meals were prepared by paying attention to the families’ cultural background [23, 49–51, 88] and in that way it was possible to show respect for their culture(s). In some studies, the duration of the intervention was tailored based on needs (supported playgroups), or school year (MEES). Interventions were generally delivered in group format and if participants were parents and children (especially youths), they had both separate and shared group sessions. In some cases, male and female participants had separate groups. In the Connect-programme, childcare services were offered during sessions to ensure parents’ participation [75, 86, 87].

The intervention providers were most often called facilitators [25, 31, 36, 44, 45, 57, 68, 98, 105], trained group leaders [58, 73–75, 85–87, 95], or peer-mentors [81, 100]. Most of these providers had some kind of cultural link to the target population and their culture, e.g. providers were bicultural or bilingual [25, 31, 98]. In some cases, the proficiency in the host country’s language was an inclusion criterion for participation [64, 73] or the intervention was delivered in two main languages of participants (for example English and Spanish) [39]. Also matching volunteers and participants based on participants’ preferred language or grouping participants based on language was done by intervention providers [54, 57]. Most often the interventions were bilingual [29, 32–34, 39, 50, 51, 57, 74, 83, 84, 99, 106, 111], and some of them also aimed to educate in language skills [47, 48, 61]. Some interventions were delivered solely in the participants’ native language [73, 75, 80, 93, 101, 107, 110] or used layman or professional interpreters [56, 58, 59, 71, 78]. It was also seen as important that providers had appropriate education and work experience. Providers’ gender was also mentioned as being an important consideration (Familias Unidas, Social Support Intervention, CAFES). In some cases, the physical location of the intervention delivery was also mentioned. In these cases, most often the intervention was delivered in communal settings, like schools [58, 73, 74, 85, 95], community-agencies [25, 31, 98] or in co-operation with local community-based organisations [57, 68]. CAPAS-intervention was delivered in local religious organisation establishments. Overall, it was seen as important to deliver the intervention in places which are neutral, feel safe and are accepted by the target population.

### Tailoring of the Interventions

Cultural tailoring was commonly used to tailor interventions for suitability in relation to target populations. Tailoring was related to the intention to participate, acceptability, adherence and to reduce dropouts. Few studies however reported on the methods used to conduct cultural tailoring and in those studies that did report on this aspect it was generally via either a community-based participatory research (CBPR) approach [25, 31, 98] or a qualitative approach [23, 43–45, 71, 83, 84, 88]. The purpose of cultural tailoring was to ensure that interventions identify the needs of the target population and is suitable from a linguistic, religious and cultural point of view. For example, Osman et al. [75] found that cultural sensitivity during intervention delivery ensured parents’ participation. Tailoring was done purely based on needs [57, 68] and gender [102, 103]. It was also highlighted in the articles that immigrant families as well as parents have unique and individual needs.

### Effectiveness of the Parent Support Programmes

Findings from the RCT studies (n = 18) were promising. Parent support programmes were effective in reducing children’s externalising [23, 84, 85, 92] and internalising problems [85, 89], attention problems [23], depression symptoms [38, 92] and traumatic stress reactions [92] when compared to the control group. Children in the intervention group reported less family arguing [92] than those in the control group. Parent support programmes were reported to improve parenting practices and skills [44, 84, 85, 89], promote positive parenting [84, 85] and reduce negative discipline or harsh punishment [84, 88]. In addition, improvements were seen in parent–child-relationship quality [88], problem-solving communication [38] and family functioning [88]. Parent support programmes have a great potential to reduce immigration-related stress [36], improve parental mental health and sense of competence in parenting [87]. One of the RCT studies did however report no effects on maternal mental distress, although the results here may have been due to the low levels of mental distress in the intervention group mothers at baseline [83].

Many of the programmes were targeted solely at families from migrant backgrounds, however, one study examined whether different target groups (migrant or non-migrant background) would benefit differently from the parent support programme. They found that migrants and non-migrants benefited equally from the parent support programme. They also found that only psychological problems in early childhood proved to be relevant in the prediction of psychological problems in adolescence, not migrant background or social status itself. [64] The investigation was conducted in Germany with 70 families with migrant backgrounds and 291 families without migrant backgrounds as a longitudinal study, where the assessments were conducted in early childhood (mean age of the children 4.2 years) and then again 10 years later in adolescence [64]. This finding highlights the importance of supporting migrant families at an early stage, since the migration event itself does not automatically mean psychological problems in children from migrant backgrounds.

### Engagement, Satisfaction and Acceptability of the Parent Support Programmes

Studies that reported on the effects of parent support programmes and which conducted some measurements on feasibility, were reported to have high retention rates and satisfaction which can be seen as indicating a level of programme feasibility [85, 92]. Suggestions on how to improve feasibility from the intervention providers include giving participants more time for rehearsal and to adopt the new skills taught in the parent support programme [85].

### Findings from the Qualitative Studies

The findings from the qualitative studies (data collected most often via focus group interviews) revealed that parents reported factors that improve participation as well as barriers to participation and challenges to carrying out the intervention. The factors that enhanced participation included motivation, incentives and trust [31]. Individual and family reasons, social reasons and fixed schedules were seen as barriers [31]. Also, parents may be more inclined to participate than adolescents [26]. Recognised challenges included the fit between intervention provider’ and parents’ expectations [73] or participants cultural background [74].

Qualitative studies reported parent perceived benefits. Parents reported positive consequences by increased positive parenting [71] and parenting competence [81], knowledge, attitudes, and behaviour [70]. Intervention targeting to fathers, increased their recognition and knowledge of mother’s depression [49]. Interventions also increased parents’ impression of positive feelings [82] and reduced loneliness and isolation [81]. One study reported that intervention increased parents’ capacity to attain education and employment [81]. Parents also recognised differences between their parenting skills and children’s’ behaviour [74] and mothers gained in terms of the ability to cope better [77].

The results of qualitative studies also revealed that interventions should be culturally sensitive and relevant [75, 80], should aim to improve parenting skills [80], communication skills [82] and that they need to include fathers in interventions if possible [49]. It was also noted that immigrant families should be viewed not in generalised terms but rather as individual units [76] and that their specific histories should be understood [42]. It is also important to listen to families [76, 78], but listening should be done carefully without the provocation of traumatic memories [78]. Also, families may have different goals in terms of the interventions [79]. Parents suggested that interventions should be delivered flexibly and include online options [72].

The role of facilitators was seen important, [68, 81] they should be respectful and collaborative [80] and know the culture and its’ values [76, 80]. Cultural tailoring and intercultural competence were thus seen as important [76].

### Findings from the Mixed Methods Studies

Findings from the mixed methods studies were similar to those of the quantitative and qualitative studies. The methods used in the mixed methods studies were pre-test-post-test design combined with individual and focus group interviews [29, 35, 61, 98, 110], analysis of demographics or home visit records, telephone surveys and questionnaires [37, 54].

In mixed methods studies, parent support programmes were reported to improve family communication (between parents and between parent and child), relationships [35, 54, 61, 98, 106, 110] and social problem-solving skills in children [35]. Parents reported reductions in adolescent behavioural problems [35, 40]. The programmes were effective or perceived to be effective in improving the mental health of both parents and children [29, 106]. Shared experiences and listening to and supporting each other in parent support groups were seen as being of paramount importance here [61]. Parenting skills, practices, self-efficacy and confidence were improved or perceived to be higher after parent support programmes [54, 61, 98, 110]. One study however reported that the parent support programme had no effect on children’s aggression [35].

### Findings from the Literature Reviews

The data search revealed only two literature reviews [4, 65]. One explored the barriers that immigrant families with children on the autism spectrum disorder (ASD) face, describing the parent support programmes used to address the barriers [65]. The other explored interventions targeted at traumatised immigrants and refugees [4].

The barriers that immigrant families with a child with ASD faced included, delayed diagnosis, difficulties in accessing services and cultural beliefs about child development. The article also presents a programme that is specifically targeted at immigrants with a child with ASD. The article included 21 articles, however, the detailed methods remained unreported [65].

The second review reported included six studies of which four reported findings from school-based interventions (targeted solely at children and adolescents) while two reported family support programmes, both of which were included in this review [93, 109]. The focus of the review by Slobodin et al. [4] was slightly different than ours since they also included studies that provided interventions in respect of children and adolescents without an intervention component relating to parents. They concluded that there is a shortage of research in this area and that is why firm conclusions cannot be made [4].

### Findings from the Quasi-Experimental, Cross-Sectional, Feasibility and Pilot Studies

The results from non-RCT quantitative studies were promising however, as expected, many of them (n = 8/13) had small sample sizes ranging from 14 to 50 parents [47, 48, 50, 99, 105, 107, 108, 112]. These small but promising studies found that parent support programmes increased family functioning and reduced child behavioural problems reported by mothers [50]. Children reported better psychological functioning, acceptance and parenting warmth after the parent support programme [50]. Parents showed empathy towards their children and gained knowledge of the alternatives to corporal punishment after taking part to the parent support programme [112]. Kindergarten children whose families took part in the Migrant Education Even Start (MEES) programme performed better in English language measures in two follow-up measurement points than the children in the control group [47, 48]. Parent support programmes had high satisfaction and engagement rates and were seen as feasible even in fragile populations or contexts and also as urgently needed [62, 105, 107]. One small pilot study reported that cultural tailoring with a facilitator from the target population’s culture was time consuming and that they faced challenges in programme implementation particularly in respect of drop-out rates [99].

In five (n = 5/13) quantitative studies the sample ranged from 85 to 408 parents [46, 62, 100, 104, 109]. Parent support programmes were found to decrease parenting stress, loneliness, and isolation [100]. By taking part in the parent support programme, parents were encouraged to find and receive social, spousal and informational support and as well as learning a number of coping strategies [100]. Those families who valued shaming in child-rearing or who were dealing with the child protection system were however less likely to perceive the parent training to be acceptable [104]. The reasons as to why drop-outs occurred from the parent support programmes included family problems or sickness in the family, travel, change in life circumstances (for example leaving the country or starting work), lack of interest, and programme burden [62]. To successfully implement a parent support programme, its content need to be interesting to the participants while the provider must have a high level of competence in leading the programme. Negative characteristics in terms of programme implementation included the deemed unsuitability of the place where the sessions were being held (for example relating to the need to travel, or not familiar/felt safe, noise, materials needed not available), too complex content or homework and too lengthy sessions [62]. Monetary compensation and providing food were seen as participation enablers. The perceived benefits of the programme included, for example, changes in social and communicational skills, resilience and wellbeing [62]. One of the studies concentrated on parents’ technological skills enabling them to help their children with their school work. The study found significant differences between the pre- and post-test in terms of parent’s technology skills and self-efficacy in helping their children [46].

The benefits of the diverse programmes reported in the included studies are summarised in Table 4.

**Table 4:** Benefits of parent support programmes to children, parents and the whole family

	Improvements	Reductions
Children and adolescents	Psychological functioning Social problem-solving skills Mental health English language skills	Behavioural problems Externalising and internalising problems Attention problems Traumatic stress reactions Depression
Parents	Parenting skills and practices Positive parenting Resilience Wellbeing Parenting self-efficacy and confidence Improved mental health* High retention rate, satisfaction and engagement Sense of parenting competence Knowledge, attitudes and behaviour Recognition of mothers’ depression Positive feelings Coping strategies Received social, spousal and informational support Parenting warmth and empathy Knowledge of the alternatives of corporal punishment Technological skills	Negative discipline Harsh punishment Loneliness and isolation Parenting stress
Family	Family communication and relationships Parent–child-relationship quality Problem-solving communication Family functioning Social skills	Family arguing Immigration related stress

*One study reported no effects on maternal mental distress

## Discussion

The aim of this study was to describe what is known about the parent support programmes that are targeted at families who are immigrants. We were primarily interested in the objectives of the programmes, their content, recruitment strategies and, ultimately, the findings in respect of these programmes.

The objectives of the interventions were to support, enhance and strengthen parenting and to improve our knowledge of positive parenting practices. Most interventions included components of positive parenting and family communication. Through improved communication, the aim was to reduce child behavioural problems or, more specifically for example, substance abuse in adolescents. A common feature of the programmes was also to provide social and peer support to families even though this was not the primary aim of the programme. Interventions were usually conducted through group-based methods. Groups provided the possibility for social integration. They also created a platform where experiences relating to the programme topics could be shared and discussions, generated by the programme topics which were often sensitive, relating to issues such as mental health, could be held in a safe environment.

On recruitment strategies, we found that the most often used recruitment strategy reported in the included studies were recruitment from the local immigrant communities and agencies/organisations. Studies reported that direct invitation with a bilingual approach and word-of-mouth invitations were considered effective [28, 36, 44, 62, 80, 84, 89, 101, 105–107]. Only one study reported using social media for recruitment [98] which could be one potential way to reach participants in the future programmes. In addition, respected and trusted community members were seen as important links between the parent support programme providers and the target participants [92].

Overall, the findings of the included studies, regardless of the design and methods, led same direction: the parent support programmes that were well planned, structured and organised were viewed as beneficial to parents and children. Both –quantitative and qualitative– results of this review indicated that the programmes had a positive influence on overall child, parent and family wellbeing as well as on parenting skills and practices. This is worth noting, since parental wellbeing is associated with children’s wellbeing [113–115] and parental mental health problems are associated with the increased use of paediatric health care services [114]. It is profitable to invest in evidence-based preventive actions, such as parent support programmes that are proven to be effective.

As the present scoping review presents, the evidence-base regarding the effective parent support programmes targeted at families who are immigrants is still being assembled, we already have the results of a systematic review, a meta-analysis and meta-regression studies suggesting that extensive cultural adaptation is not necessary for the successful transportation of evidence-based parent support programmes [116, 117]. We have also learned from a recent study that a group-based parent support programme which is used worldwide, incredible years, had similar effects on child behavioural problems regardless of the family ethnic minority status [118]. These results suggest that when selecting the parent support programme for families who are immigrants, it may be more important to choose a well-studied intervention, with evidence of effectiveness, than to create resource consuming cultural adaptation actions [116–118]. This is however somewhat contradictory to our results, since many articles specifically emphasised the importance of need-based and culturally relevant programmes. Our explanation for this is that the need-based approach and culturally adapted programmes are important in terms of recruiting, engaging and acceptance of the programmes, even though the programme’s ultimate effectiveness relates to its well-structured and evidence-based content. Based on our findings, identifying the needs of the target group and cultural tailoring were highly important in terms of programme acceptability and results*.*

The cultural tailoring element was an important part of the interventions because cultural sensitivity and use of one’s own language ensured participation. It was also seen as important to understand the immigrant families’ background and that is why facilitators should understand the culture. In addition, religion is an important part of many cultures [119] and, as such, it is crucial to understand the culture also from this perspective, as religion can influence peoples’ actions and reactions, for example help-seeking or receiving help [120] or even parenting [121]. Nevertheless, the definitions and descriptions in respect of cultural tailoring remained either unclear or overly narrow in most of these studies.

We would therefore like to suggest that cultural tailoring should be seen as a bi-directional process including cultural tailoring from the perspective of immigrants’ cultural background, but also information about new host country’s cultural manners and procedures. This kind of cultural tailoring and social integration should be based on evidence-based interventions. As presented in the evidence-based Ladnaan -programme [75, 86, 87], the programme included information about the host-country’s society, the social- and health care systems and legislation in the regulated intervention, as it was deemed important to improve the knowledge of immigrant parents from this perspective. This may help them to understand better, why particular things are done as they are done, e.g. child protection actions and how the legislation of their new country deals with physical discipline. Increasing awareness of the fact that the physical punishment of children is prohibited by law, it may be possible to prevent harsh discipline and physical conflicts within the family. The preventive perspective is worth noting, since youth violence is also lower in countries with a complete ban on corporal punishment [122]. Parents do however need skills in terms of understanding alternative approaches to corporal punishment in dealing with challenging situations with their children.

All in all, the uncertainty in balancing between using a structured and highly regulated intervention and need-based and cultural tailoring, clearly demands that this area requires further investigation in terms of programmes targeted at immigrant families. Additionally, one other thing also caught our attention: The majority of the studies were conducted in North America with Latin American populations. Considering the European migrant crisis which began in 2014–2015 [123], the number of studies conducted in Europe was quite low. Also, there were only one study reporting a parent support program in initial registration centre [78] and of specific age groups, programs targeted to families with small children were limited. In the future, it would perhaps also be relevant to investigate how digitalisation and technology could be better utilised in parent support programmes.

### Strengths and Limitations

It must be noted that these findings are summarised from different parent support programmes with heterogenous participants and thus that the findings reported here should be interpreted with caution. In addition, a quality appraisal of these studies has not been conducted in the context of the present study, although this is something that is not generally considered necessary in scoping reviews [18].

The study selection and data charting were conducted by two independent reviewers which is considered to strengthen the methodological quality of the current study. We did not have any time or language restrictions in our search strategy and we also covered the ‘grey’ literature from OpenGray, thus minimising the risk of publication bias. We did use a language translator with the German language articles. Articles in other languages (Spanish, Dutch, French. see Fig. 1) were not available as full-texts, so they were excluded at the full-text phase. One of the studies published in German was the only study that were conducted at the initial registration centre [78] and thus might provide the context for arguments over the need to begin evidence-based parent support at the earliest phase possible. This previously mentioned article also highlights the importance of including studies in reviews with no language restrictions.

## Conclusion

To conclude, parent support programmes for families who are immigrants are essential to promote better parental practices and families’ overall wellbeing. When planning parent support programmes for families who are immigrants there are many applicable and effective interventions to be exploited. Nevertheless, it is important to tailor interventions to be culturally sensitive for better recruitment rates, engagement and acceptance. This cultural tailoring should include the tailoring of linguistical needs as well as cultural manners, beliefs and traditions during the planning and implementation of an evidence-based intervention. Furthermore, it is important to share information about the social- and welfare system, legislation and policy of the new host country, because families –especially parents– who are immigrants, need this type of information and knowledge in order to better understand the actions of public officials. Interventions can be delivered in many ways; the most important thing is to consider the target population, their motives and needs in order to achieve the best results and benefits possible.

## Supplementary Information

Below is the link to the electronic supplementary material.

Supplementary file1 (DOCX 15 kb)

Supplementary file2 (DOCX 48 kb)
