# Supplementary material for: Parent Support Programmes for Families Who are Immigrants: A Scoping Review
Source: J Immigr Minor Health. 2021 Mar 26;24(2):506–25. doi: 10.1007/s10903-021-01181-z (PMC8854318; doi:10.1007/s10903-021-01181-z)
Supplement: Supplementary file 1 — Supplementary file1 (DOCX 15 kb) [file 10903_2021_1181_MOESM1_ESM.docx]

**Appendix 1** Search strategy

1.Search strategy

**a. PubMed**

**S1** ("Emigrants and Immigrants"[Majr] OR "Emigration and Immigration"[Majr] OR "Refugees"[Majr]) OR ***Title*** (immigrant* OR immigrat* OR migrant* OR migrat* OR emigrant* OR emigrat* OR nomad* OR foreigner* OR “asylum seeker*” OR refugee* OR “stateless person*” OR noncitizen* OR non-citizen* OR outsider* OR newcomer* OR new-comer* OR incomer* OR “newly arrived” OR “new arrival*” OR “recent entrant*” OR non-national OR non-native* OR “naturalized citizen*” OR settler* OR expatriate* OR “displaced person” OR “displaced people”)

**S2** ("Parenting"[Majr] OR "Parents"[Majr] OR "Caregivers"[Majr] OR "Parent-Child Relations"[Majr] OR “Family”[Majr]) OR ***Title*** (parent OR parents OR parenting* OR mother* OR father* OR family* OR families* OR parent-child* OR carer* OR caregiver* OR guardian*)

**S3** ("Social Support"[Majr] OR "Health Promotion"[Majr] OR "Counseling"[Majr]) OR ***Title*** (support* OR program* OR training* OR intervention* OR counsel* OR promot*) OR (“parent support” OR “parenting support” OR “parent program” OR “parenting program” OR “parent support program” OR “parenting support program” OR “parent training” OR “parenting training” OR “parent intervention” OR “parenting intervention” OR “parent education” OR “parenting education” OR “family support” OR “family program” OR “family training” OR “family intervention” OR “family education”)

**b. Web of Science, EBSCO-databases**

**S1** ***Title*** (immigrant* OR immigrat* OR migrant* OR migrat* OR emigrant* OR emigrat* OR nomad* OR foreigner* OR “asylum seeker*” OR refugee* OR “stateless person*” OR noncitizen* OR non-citizen* OR outsider* OR newcomer* OR new-comer* OR incomer* OR “newly arrived” OR “new arrival*” OR “recent entrant*” OR non-national OR non-native* OR “naturalized citizen*” OR settler* OR expatriate* OR “displaced person” OR “displaced people”)

**S2** ***Title*** (parent OR parents OR parenting* OR mother* OR father* OR family* OR families* OR parent-child* OR carer* OR caregiver* OR guardian*)

**S3** ***Title*** (support* OR program* OR training* OR intervention* OR counsel* OR promot*) OR (“parent support” OR “parenting support” OR “parent program” OR “parenting program” OR “parent support program” OR “parenting support program” OR “parent training” OR “parenting training” OR “parent intervention” OR “parenting intervention” OR “parent education” OR “parenting education” OR “family support” OR “family program” OR “family training” OR “family intervention” OR “family education” OR “health promotion”)

**c. Medic**

**S1** ***Te/Ot/As/Ti*** (immigra* refugee* asylum* maahanmuut* pakolais* turvapaik*)

**S2** ***Te/Ot/As/Ti*** (parent* mother* father* vanhem* äidi* isä*)

**S3** ***Te/Ot/As/Ti*** (support* program* interventio* tukem* koulutu* neuvon*)

**d. Cochrane**

**S1** ("Emigrants and Immigrants"[MeSH] OR "Emigration and Immigration"[MeSH] OR "Refugees"[MeSH]) OR ***Title*** (immigrant* OR immigrat* OR migrant* OR migrat* OR emigrant* OR emigrat* OR nomad* OR foreigner* OR “asylum seeker*” OR refugee* OR “stateless person*” OR noncitizen* OR non-citizen* OR outsider* OR newcomer* OR new-comer* OR incomer* OR “newly arrived” OR “new arrival*” OR “recent entrant*” OR non-national OR non-native* OR “naturalized citizen*” OR settler* OR expatriate* OR “displaced person” OR “displaced people”)

**S2** ("Parenting"[MeSH] OR "Parents"[MeSH] OR "Caregivers"[MeSH] OR "Parent-Child Relations"[MeSH] OR “Family”[MeSH]) OR ***Title*** (parent OR parents OR parenting* OR mother* OR father* OR family* OR families* OR parent-child* OR carer* OR caregiver* OR guardian*)

**S3** ("Social Support"[MeSH] OR "Health Promotion"[MeSH] OR "Counseling"[MeSH]) OR ***Title*** (support* OR program* OR training* OR intervention* OR counsel* OR promot*) OR (“parent support” OR “parenting support” OR “parent program” OR “parenting program” OR “parent support program” OR “parenting support program” OR “parent training” OR “parenting training” OR “parent intervention” OR “parenting intervention” OR “parent education” OR “parenting education” OR “family support” OR “family program” OR “family training” OR “family intervention” OR “family education”)

2. Search queries

**a. PubMed**

((((("Emigrants and Immigrants"[Majr] OR "Emigration and Immigration"[Majr] OR "Refugees"[Majr])) OR (immigrant*[Title] OR immigrat*[Title] OR migrant*[Title] OR migrat*[Title] OR emigrant*[Title] OR emigrat*[Title] OR nomad*[Title] OR foreigner*[Title] OR "asylum seeker*"[Title] OR refugee*[Title] OR "stateless person*"[Title] OR noncitizen*[Title] OR non-citizen*[Title] OR outsider*[Title] OR newcomer*[Title] OR new-comer*[Title] OR incomer*[Title] OR "newly arrived"[Title] OR "new arrival*"[Title] OR "recent entrant*"[Title] OR non-national[Title] OR non-native*[Title] OR "naturalized citizen*"[Title] OR settler*[Title] OR expatriate*[Title] OR "displaced person"[Title] OR “displaced people”[Title]))) AND ((("Parenting"[Majr] OR "Parents"[Majr] OR "Caregivers"[Majr] OR "Parent-Child Relations"[Majr] OR "Family"[Majr])) OR (parent[Title] OR parents[Title] OR parenting*[Title] OR mother*[Title] OR father*[Title] OR family*[Title] OR families*[Title] OR parent-child*[Title] OR carer*[Title] OR caregiver*[Title] OR guardian*[Title]))) AND (((("Social Support"[Majr] OR "Health Promotion"[Majr] OR "Counseling"[Majr]) OR (support*[Title] OR program*[Title] OR training*[Title] OR intervention*[Title] OR counsel*[Title] OR promot*[Title]))) OR (“parent support” OR “parenting support” OR “parent program” OR “parenting program” OR “parent support program” OR “parenting support program” OR “parent training” OR “parenting training” OR "parent intervention" OR "parenting intervention" OR “parent education” OR “parenting education” OR “family support” OR “family program” OR “family training” OR “family intervention” OR "family education"))

**b. Web of Science**

TI=(immigrant* OR immigrat* OR migrant* OR migrat* OR emigrant* OR emigrat* OR nomad* OR foreigner* OR "asylum seeker*" OR refugee* OR "stateless person*" OR noncitizen* OR non-citizen* OR outsider* OR newcomer* OR new-comer* OR incomer* OR "newly arrived" OR "new arrival*" OR "recent entrant*" OR non-national OR non-native* OR "naturalized citizen*" OR settler* OR expatriate* OR "displaced person" OR "displaced people") AND TI=(parent OR parents OR parenting* OR mother* OR father* OR family* OR families* OR parent-child* OR carer* OR caregiver* OR guardian*) AND ((TI=(support* OR program* OR training* OR intervention* OR counsel* OR promot*) OR ALL=("parent support" OR "parenting support" OR "parent program" OR "parenting program" OR "parent support program" OR "parenting support program" OR "parent training" OR "parenting training" OR "parent intervention" OR "parenting intervention" OR "parent education" OR "parenting education" OR "family support" OR "family program" OR "family training" OR "family intervention" OR "family education" OR "health promotion")))

*EBSCO-databases*

**c. Academic Search Premier (EBSCO)**

**d. CINAHL (EBSCO)**

**e. ERIC (EBSCO)**

**f. PsycINFO (EBSCO)**

TI ( immigrant* OR immigrat* OR migrant* OR migrat* OR emigrant* OR emigrat* OR nomad* OR foreigner* OR “asylum seeker*” OR refugee* OR “stateless person*” OR noncitizen* OR non-citizen* OR outsider* OR newcomer* OR new-comer* OR incomer* OR “newly arrived” OR “new arrival*” OR “recent entrant*” OR non-national OR non-native* OR “naturalized citizen*” OR settler* OR expatriate* OR “displaced person” OR “displaced people” ) AND TI ( parent OR parents OR parenting* OR mother* OR father* OR family* OR families* OR parent-child* OR carer* OR caregiver* OR guardian* ) AND ((TI ( support* OR program* OR training* OR intervention* OR counsel* OR promot* ) OR TX ( “parent support” OR “parenting support” OR “parent program” OR “parenting program” OR “parent support program” OR “parenting support program” OR “parent training” OR “parenting training” OR "parent intervention" OR "parenting intervention" OR “parent education” OR “parenting education” OR “family support” OR “family program” OR “family training” OR “family intervention” OR “family education” OR “health promotion” )))

**g. Medic**

(immigra* refugee* asylum* maahanmuut* pakolais* turvapaik*) AND (parent* mother* father* vanhem* äidi* isä*) AND (support* program* interventio* tukem* koulutu* neuvon*) [Te/Ot/As/Ti]

**h. Cochrane**

**S1** MeSH-terms

**#1** Emigrants and Immigrants

**#2** Emigration and Immigration

**#3** Refugees

**#4 Search: #1 OR #2 OR #3**

**#5** immigrant* OR immigrat* OR migrant* OR migrat* OR emigrant* OR emigrat* OR nomad* OR foreigner* OR “asylum seeker*” OR refugee* OR “stateless person*” OR noncitizen* OR non-citizen* OR outsider* OR newcomer* OR new-comer* OR incomer*

“newly arrived” OR “new arrival*” OR “recent entrant*” OR non-national OR non-native* OR “naturalized citizen*” OR settler* OR expatriate* OR “displaced person” OR “displaced people”

**#6 Search: #4 OR #5**

**S2** MeSH-terms

**#7** Parenting

**#8** Parents

**#9** Caregivers

**#10** Parent-Child Relations

**#11** Family

**#12 Search: #7 OR #8 OR #9 OR #10 OR #11**

**#13** parent OR parents OR parenting* OR mother* OR father*

family* OR families* OR parent-child* OR carer* OR caregiver* OR guardian*

**#14 Search: #12 OR #13**

**S3** MeSH-terms

**#15** Social Support

**#16** Health Promotion

**#17** Counseling

**#18** Search: #15 OR #16 OR #17

**#19** support* OR program* OR training* OR intervention* OR counsel* OR promot*

“parent support” OR “parenting support” OR “parent program” OR “parenting program” OR “parent support program” OR “parenting support program” OR “parent intervention” OR “parenting intervention” OR “parent training” OR “parenting training” OR “parent education” OR “parenting education” OR “family support” OR “family program” OR “family training” OR “family intervention” OR “family education” OR “health promotion”

**#20 Search: #18 OR #19**

**#21 Final search: #6 AND #14 AND #20**
