# Supplementary material for: Parent Support Programmes for Families Who are Immigrants: A Scoping Review
Source: J Immigr Minor Health. 2021 Mar 26;24(2):506–25. doi: 10.1007/s10903-021-01181-z (PMC8854318; doi:10.1007/s10903-021-01181-z)
Supplement: Supplementary file 2 — Supplementary file2 (DOCX 48 kb) [file 10903_2021_1181_MOESM2_ESM.docx]

**Appendix 2** Data charting table

| **Author, Year, Country** | **Aim of the study** | **Methods** | **Study population / Target population** | **Key findings** |
| --- | --- | --- | --- | --- |
| Allen et al. 2012, USA | To report the study protocol of a study which aims to assess the efficacy of a family-skills training intervention to prevent substance use intentions in Latinx youth. | Study protocol of CBPR, Targeted sample size n = 336 families | Latinx youth and their parents | - |
| Annan et al. 2017, USA/ Thailand | To examine the effectiveness of a parenting skills intervention on mental health outcomes. | RCT, n = 479 families | Burmese caregivers and children aged 7–15 years | Significant reductions in externalizing problems and child attention problems in IG compared with CG. Significant increase in prosocial protective factors in IG compared with CG. |
| Azziz-Baumgartner & Wilson 2009, USA | To describe issues to consider and discuss application of a process to adapt the Familias Fuertes (FF) intervention for Latinx immigrant families in USA. | Qualitative, focus group, questionnaire (acculturation Scale), n = 7 adolescents, n = 10 parents | Latinx immigrant families | Parents had a higher Latin orientation than the adolescents. Most important issues ranked by youth & parents: *Lack of extended family & support *Communication and language problems *Lack of time with family *Cultural differences *peer pressure leading to risk for drug abuse, teen pregnancy, and other problems. *Discrimination. |
| Bacallao & Smokowski 2005, USA | To conduct a review of bicultural skills training interventions and introduce a refined model of bicultural skills training for Latinx immigrant families. | review */ theoretical article | Latinx immigrant families | Bicultural skills training interventions have shown promising initial results on a variety of outcomes. Based on literature and local data collection Entre Dos Mundos (Between Two Worlds) intervention was developed and is presented in the article. |
| Ballard & Forgatch 2017, USA | To test the feasibility of implementing an adapted evidence-based parenting intervention for contexts of trauma and relocation stress. | Qualitative, Ethnographical interviews and structured assessments, n = 11 | Karen refugees from Burma | Participants reported changes in their teaching, directions, emotional regulation, discipline, and child compliance. Children reported changes in positive parent involvement and decrease in mental health symptoms. |
| Bernhard 2010, Canada | To report the development of theoretically based interventions for newcomer (immigrant)parents. | * | Latin American parents of children aged 4-8 | The article provides information and experiences of interventions that aim to engage immigrant parents in their children’s education. |
| Betancourt et al. 2019, USA | To conduct a pilot feasibility and acceptability trial of the home-visiting Family Strengthening Intervention for refugees (FSI-R). | CBPR, n = 80 families (n = 152 children, n = 105 caregivers) families were randomized to FSI-R or care as usual. | Somali Bantu and Bhutanese caregivers and children | The retention rate of 82.50% indicates high feasibility, and high reports of satisfaction (81.50%) indicate community acceptance. Reduced traumatic stress reactions in IG children. Caregivers reported fewer child depression symptoms compared with CG. Bhutanese IG children reported reduced family arguing and fewer depression symptoms and conduct problems by parent report compared with CG. |
| Bjørknes & Manger 2013, Norway | To assess the intervention effects of Parent Management Training—Oregon Model (PMTO) on maternal-parent practices and child behavior. | RCT, n = 96 mothers and their children aged 3–9 years were randomized to PMTO or WLC | Mothers from Somalia and Pakistan and their children aged 3–9 years | PMTO was effective in enhancing parent practices, with a decrease in harsh discipline and an increase in positive parenting. Reductions in mother reported child conduct problems in IG. |
| Bjørknes et al. 2015, Norway | To assess the intervention effects of Parent Management Training — Oregon Model (PMTO) on maternal mental distress. | RCT, n = 96 mothers and their children aged 3–9 years were randomized to PMTO or WLC | Mothers from Somalia and Pakistan and their children aged 3–9 years | Low levels of mental distress at enrollment in intervention among the sample. PMTO was not effective in alleviating maternal mental distress. |
| Cowell et al. 2000, USA | To develop and test a Mexican American problem-solving (MAPS) home-and school-based school nursing intervention. | Mixed methods, one group pre-test-posttest design (n = 18 mothers, n = 44 children), and focus groups (n = 9 mothers) | Mexican immigrant families | Mothers and children had statistically significant improvement in mental health scores after intervention. Mothers felt supported by the home visiting school nurse, and stated that the after-school program was beneficial to their children. |
| Cwikel et al. 2018, Israel | To evaluate the Mom to Mom (M2M) program in Israel. | Mixed methods, (1) Analysis of demographics (n = 440), (2) A telephone survey (n = 51), (3) questionnaire (n = 137) | (Immigrant) mothers in Israel | Demographics of mothers in M2M: first time mothers, with a high rate of perinatal complications (54.4%), positive Edinburgh Post-Natal Depression Scale scores (38.7%).  Main reasons for participation: being an immigrant and low income. Intervention improved self-confidence, parenting skills and communication with the partner. |
| Dababnah et al. 2018, USA (Turkey) | To test the feasibility and acceptability of an intervention developed specifically for children with autism spectrum disorder affected by trauma. | Qualitative, interviews after intervention completion, n = 9 parents, n = 11 teachers | Syrian refugee women and teachers | All participants were satisfied with program content. Challenges included applying skills to nonverbal children. Participants’ recommendations for program improvement: a need for services outside urban areas, flexible program delivery methods, including online options. |
| Dumka et al.2007, USA | To describe critical participatory principles and practices used in a successful partnership between university and a public elementary school district in intervention (the Puentes program) and study implementation. | CBPR* | Mexican origin families, Puentes research team, school administrators and staff | School and research team had highly valued mutual goal and resource exchange. Determining and agreeing on an appropriate scope for the partnership, listening, reciprocal learning, and power sharing was important. |
| Friedrich & Smolka 2012, Germany | To classify family education and parent education programs in Germany by employing a multi-dimensional scheme. | Review* (studies only from Germany were included) | Review study population: refugees with under 6 years old children. | Most programs produce positive short-term effects or experiences/feedback and have a low-threshold access. Positive results for the interaction/communication between mother and child, positive cognitive changes in children, and better skills in German language. Programs that had effects to parents, had effects in children. Program acceptability was mostly high. However, group-based modules had discrepancy in results about acceptance. Home visits were seen positive. Conclusions about long-term effects cannot be made. |
| Garcia Huidobro et al. 2019, USA | To evaluate an adaptive recruitment intervention to Padres Informados Jovenes Preparados (PIJP) program and to evaluate the intervention satisfaction. | Mixed methods, pre-post design (questionnaires), interviews and process evaluations, n = 15 immigrant Latinx families with 10–14-year-old child | Latinx families | 73% of two-parent families enrolled with both parents.  Most participants completed 75% or more of the intervention. Fathers were more likely to use the one-to-one component of the intervention than mothers. Participants perceived positive changes in their parental self-efficacy, parenting practices, and relationships. |
| Garcia Huidobro et al. 2016, USA | To study factors associated with attendance and nonattendance in parent support program. | Qualitative, n = 76 parents in feedback discussions, n = 10 in-depth interviews | Parents (mostly Mexican mothers) and facilitators who delivered the program | Factors that contributed to participation: motivation, incentives (e.g. food and childcare), and trusted facilitators. Barriers to participation: individual and family reasons, sociocultural reasons (e.g., community and cultural beliefs), and fixed schedules. |
| German 2008, UK | To explores how educational psychologists (EP) can promote the emotional wellbeing and resilience of refugee parents. | Review/Report/Case examples* | Somali parents, Kosovon Albanian and Turkish Kurd women | Case (1) Referrals of Somali pupils to the school EP decreased. The major themes raised by the Somali parents were: lack of understanding about the school system; inability to help their children due to linguistic barriers. Case (2) The key to the success of the project was co-working with interpreters. Case (3) Through individual discussions (before the group sessions), a number of referrals/links were made to appropriate agencies for further support, including adult mental health. Women found the social support of the groups beneficial. |
| Gonzalez 2017, USA | To describe three stages in a community-based college knowledge educational outreach program for Latinx parents (needs assessment, implementation and evaluation). | CBPR*, focus groups n = 27 parents | Latinx parents | Negative experiences were shared by parents when trying to interact with school personnel. Also, language barriers were present. Comments on the program: participants experienced the workshop informative and beneficial, advertisement and the number of meetings could be increased, Hispanic/Latinx parents could be reached out from work places, and by sending flyers home with students. |
| Gonzalez et al. 2013, USA | To review literature and describe culturally appropriate strategies for school counselors to work with Latinx population. | Review/Report/Case example* | Latinx parents | Latinx parents have constraints to their involvement in children’s education, including family-related factors, cultural expectations of their role, and barriers to receiving or acting on invitations from school personnel. |
| Guo & Gray 2017, Australia | To explore the perspectives of bilingual playgroup leaders from the Victorian Cooperative on Children’s Services for Ethnic Groups (VICSEG). | Qualitative, focus group & questionnaires, n = 7 | Playgroup leaders from different cultural backgrounds | Important: appreciating and understanding the challenges of parenting in a new country, building parents’ confidence, building children’s confidence and access to early learning experiences, and providing broader social and local agency connections. |
| Hendrickson et al. 2008, USA | To better understand pediatric home injury risk in immigrant Hispanic families in Texas and New Mexico. | RCT, pretest-posttest intervention study, questionnaires, n = 60 parents | Hispanic families | Recruitment site, depression, and the number of preschool-age children in the home emerged as significant predictors of child hazards. The intervention likely reduced maternal depression. |
| Ho et al. 2012, USA | To assess parent training acceptability and explore clinical and cultural factors  that may be associated with acceptability. | Quantitative, cross-sectional, n = 145 parents with 4–17-year old children | Chinese immigrant parents | Positive reinforcement techniques were acceptable, and more likely to be supported than punishment-based techniques. Parents who endorsed the Chinese child-rearing value of shaming or were less likely to find training acceptable. Families with prior CPS involvement rated training as less acceptable. |
| ISRCTN22321773 (2019), Holland (Lebanon) | To test the effectiveness of the Caregiver Support Intervention (CSI) among Syrian refugee families in northern Lebanon. | Study registration of RCT, questionnaires, target sample size: n = 480 Syrian parents with 3–12-year old child | Syrian refugee families | - |
| ISRCTN33665023 (2019), Holland (Lebanon) | To assess the effectiveness of evaluation methodology, prior to conducting a fully powered RCT of the Caregiver Support Intervention (CSI) with Syrian refugees in North Lebanon. | Study registration of a pilot RCT, questionnaires & focus groups, target sample size: n = 72 Syrian parents with 3–12-year old child | Syrian refugee families | - |
| Javier et al. 2019, USA | To describe lessons learned from using the Matching Model of Recruitment to recruit 215 Filipinos to participate in RCT of a video aimed at increasing enrollment in the Incredible Years® Parent Program. | Descriptive* | Filipino families | Important in recruitment: *match between goals of parents, grandparents and the research community, *ensuring confidentiality, *gaining trust *understanding the perspectives of ethnic minority communities. |
| Kim et al. 2010, USA | To evaluate the delivery of a parenting program to 17 Korean immigrants for cultural and linguistic appropriateness and usefulness regarding recruitment, retention, program content, and delivery methods. | Qualitative, focus group, n = 17 parents with 3–8-year old child. | Korean immigrant parents | Themes identified included fit between the parents’ desire and what the program offered, effective recruitment and retention strategies, program content and videotapes. |
| Kim et al. 2016, USA | To explore the perceptions of Korean American parents on effective parenting strategies taught in a standardized U.S. parenting program | Qualitative, individual interviews with semi-structured open-ended questions, n = 22 parents | Korean immigrant parents | Themes that emerged were: effective parenting strategies (impact on parents and children). Findings indicated that Korean American parents felt the program was based on Western parenting strategies but they were effective and made a difference in their parenting skills and children’s behavior. |
| Knox et al. 2011, USA | To evaluate the effectiveness of the evidence-based program, Families and Schools Together (FAST) with Latinx families. | Mixed methods, questionnaires n = 282 parents, n = 282 children, and focus group, n = 10 parents | Latinx families | No differences on aggression between IG and CG. Significant improvements in social problem-solving skills in IG children. Focus groups: Intervention helped in communication with their children. The greatest (perceived) effect was on the behavior of their older children. |
| Lau et al. 2010, USA | To describe the application of group parent training in two Chinese immigrant families. | Report of 2 cases | Chinese immigrant families | Two immigrant families referred to treatment by CPS for suspected maltreatment. Both cases illustrate the value of initial engagement processes to overcome cultural misgivings about parent training as well as mistrust and stigma associated with involuntary treatment. Parents can be effectively engaged in group parent training even when they are not in treatment voluntarily. |
| Lau et al. 2011, USA | To study the efficacy and implementation outcomes of a culturally responsive parent training (PT) program. | RCT, n = 54 parents with 5–12-year old children. Qualitative feedback from group leaders | Chinese American | Retention and engagement were high. Intervention was effective in reducing negative discipline, increasing positive parenting, and decreasing child externalizing and internalizing problems. Qualitative impressions from group leaders suggested that slower pacing and increased rehearsal of skills may improve efficacy for immigrant parents unfamiliar with skills introduced in PT. |
| Leijten et al. 2016, The Netherlands | To test whether lower levels of problem perception diminish parenting intervention (Incredible Years) effects to reduce disruptive child behavior. | RCT, n = 136 mothers of 3–8-year-olds | Dutch, Moroccan and Turkish mothers with their children | Maternal problem perception did not affect parenting intervention effectiveness. |
| Lopez-Zeron et al. 2019, USA | To contrast the differential efficacy of two culturally adapted versions of the GenerationPMTO intervention. | RCT, n = 103 families (n = 189 individuals) of 4–12-year-olds; focus group interviews | Mexican origin immigrant families | Culturally adapted parenting interventions have a potential to reduce the impact of immigration-related stressors that negatively impact Mexican-origin immigrant parents. |
| McNaughton et al. 2010, USA | To describe the types of problems Mexican immigrant women revealed to nurses in a school-based home visiting program in Chicago. | Mixed methods, n = 121 mothers | Mexican mothers | Nine categories of problems were developed from the data. Most frequently identified problems: family health concerns and access to health care, parenting and financial concerns, acculturation, mother's mental health, alcohol abuse, intimate partner violence and neighborhood violence. |
| McNaughton et al. 2014, USA | To assess feasibility of the adapted intervention (Mission Possible) in a school setting. | Qualitative, focus groups, n = 27 mother–child dyads | Mexican immigrant mothers | Findings of this study supported feasibility. Mothers and children expressed satisfaction with the intervention. Mothers stated that they enjoyed the intervention and wanted to attend if future groups were offered. |
| McNaughton et al. 2015, USA | To assess the efficacy of an adapted mother–child communication intervention for Latinx immigrants. | RCT, n = 53 mother–child dyads | Latinx immigrant mothers and their fourth- to sixth-grade children | Children in the IG reported significant improvements in problem-solving communication, health self-concept, and decrease in depressive symptoms and when compared to CG. Mothers in the IG reported significant improvement in family conflict compared to CG. |
| Measham et al. 2014, Canada | To highlight models of mental health care for refugee children and their families, focusing on collaborative care with primary care providers. | Report of 3 cases* | African and Asian children (8–12-year old) | Holistic approach addressing biopsychosocial needs is often recommended both for assessment and treatment of refugee children. Primary care providers have a key role in detecting, assessing, and proposing treatment to vulnerable children. |
| Nagoshi et al. 2018, USA | To pilot a parenting skills curriculum to prevent adolescent substance use among Burmese families. | Pilot study, pretest-posttest survey, focus group, n = 14 mothers | Burmese refugee families | Burmese facilitator and translator were needed to help adapt  the curriculum. Curriculum adaptation was time consuming (consideration of Burmese cultural norms and practices regarding parenting, education, and religion). There were also challenges with curriculum implementation (e.g. drop-outs, illiterate participants). |
| NCT02829086, 2016, USA | To measure if the interventions improve the well-being of individuals and families within the refugee and immigrant population served by USCRI. | Study registration of RCT with 6 months follow-up | Refugee and immigrant population served by USCRI. | - |
| NCT03040154, 2017, USA | To evaluate the outcomes of engagement intervention aimed at promoting the participation in an evidence-based preventive parenting intervention. | Study registration of RCT with 6 months follow-up, target sample size n = 215 | Filipino parents and grandparents | - |
| Niewboer & van't Rood 2016, The Netherlands and Sweden | To present a feasible alternative to current language-oriented curricula for civic integration. | Mixed methods, interviews and focus group, n = 16 participants | Migrant mothers of Berber and Arabic origin | Mothers reported being less shy and more comfortable with themselves. They felt more able to express their needs and expectations, and to defend their personal interests. They also gained parenting skills and skills related to how to communicate effectively to prevent escalation. They had become less impulsive in their reactions towards others. The group members developed a bond by sharing experiences, listening and supporting each other, and accepting and respecting each other’s personalities. |
| Osman et al. 2017, Sweden | To evaluate a culturally tailored parenting support program (Ladnaan) to determine its effectiveness on children’s emotional and behavioral problems. | RCT, n = 120 parents | Somali born parents | The overall findings were: the intervention reduced behavior problems in immigrant children two months after the intervention. |
| Osman et al. 2019, Sweden | To describe Somali parents’ experiences of how a culturally sensitive programme affected their parenting. | Qualitative study, semi-structured interviews, n = 50 parents | Somali born parents | Parenting programmes should be tailored to the specific needs of the participants and cultural sensitivity should be factored into programmes to attract immigrant parents. The overarching category, a light has been shed: Two subcategories, confidence in parenting and being emotionally aware and available, emerged from the inductive analysis. The subcategory of, cultural sensitivity in the parenting programme, emerged from both the inductive and deductive analysis. |
| Osman, Salari et al. 2017, Sweden | To evaluate the effectiveness of a culturally tailored parenting support programme on Somali-born parents’ mental health and sense of competence in parenting. | RCT, n = 120 parents with children aged 11–16 years | Somali born parents | A culturally tailored parenting support programme improved the mental health and sense of competence in parenting of Somali-born parents 2 months after the intervention. |
| Pantin et al. 2003, USA | To study efficacy of Familias Unidas in promoting protection against and reducing risk for adolescent behavior problems. | Mixed methods, RCT, n = 167 adolescents and their families | Hispanic immigrant families | Families participating in Familias Unidas reported consistent reductions in adolescent behavior problems. |
| Pantin, Schwartz et al. 2003, USA | To discuss the challenges that Hispanic  immigrant families face, to review the consequences, and to outline a preventive intervention (theoretical background and methods). | Theoretical article (intervention description) | Hispanic immigrant families | The combination of a multidimensional focus, parent empowerment, and culturally appropriate intervention activities may help to address the risks associated with immigration, financial difficulties, and acculturation. Key intervention principles of Familias Unidas were presented. |
| Paris 2008, USA | To describe the experiences of Latinx immigrant participants in a home visiting program to understand their needs and perceptions of the intervention. | Qualitative, interviews, n = 14 | Latinx immigrants | Three themes: (a) beginning the immigrant journey (reasons for immigration, suffering during the travel); (b) life in a new country (isolation, depression, children left behind, and personal strengths and supports); and (c) the helpfulness of the home visitors in supporting adaptation and resiliency (emotional support, case management/advocacy, translation, education, and friendship). |
| Parra Cardona et al. 2019, USA | To present a model of intervention delivery utilized in the dissemination of culturally adapted versions of the evidence-based intervention known as GenerationPMTO. | Theoretical article (introducing a model) | Latinx immigrant parents | Process of change: enhancing parenting practices. Model includes: Embracing the parenting dream, becoming aware (giving good directions), promoting strengths and nurturing parent–child relationships, teaching children and youth to live within boundaries (monitoring and supervision), and embracing culture. |
| Parra Cardona et al. 2009, USA | To explore Latinx immigrant parents’ perceptions about the relevance of adapting a parenting intervention. | Qualitative, focus groups, n = 83 parents | Latinx immigrant parents | Categories: Interventions should aim to improve parenting skills, interventions should be culturally relevant and managed with respect, educators need to be respectful and collaborative, and awareness of relevant Latin cultural values is important. |
| Parra Cardona et al. 2012, USA | To describe the results of the initial feasibility and cultural acceptability pilot study of a parent training program. | A pilot study, n = 24 parents | Latinx immigrant parents | High engagement and retention were achieved in both adapted interventions with 91% of participants attending at least 9 of the 12 curriculum sessions. Parents experienced high levels of satisfaction with both interventions. |
| Parra Cardona et al. 2017, USA | To compare the impact of two differentially culturally adapted versions of the evidence-based parenting intervention (PMTO). | RCT with 6 months follow-up, n = 103 families (n = 190 individual parents) | Latinx immigrant parents | Statistically significant improvements on parenting skills at 6-month follow-up in both adapted interventions, when compared to the control condition. |
| Parra Cardona, López-Zerón et al. 2017, USA | To reflect on the process of change of Latinx immigrant parents exposed to a culturally adapted parenting intervention. | Theoretical article / process description* | Latinx immigrant parents | - Successful engagement of parents in the first session is essential - joining skills of the intervention delivery team are essential - location that is associated safe and familiar - promoting an initial sense of group cohesion (dinner, ice braking activities)   It is imperative for professionals to have an understanding of the process of change that parents experience as they enhance their parenting skills and incorporate new practices. Ultimately, the ability to grow in the midst of adversity is about embracing existing strengths and nurturing the human experience that impacts everyone involved in the journey. |
| Pejic et al. 2016, USA | To highlight the critical features of effective family support programs for refugee families. | Report / case example * | Somali Parents | The article presents a community-based approach that targets family interventions and services through a preventive, family systems ecological framework. |
| Pejic et al. 2017, USA | To identify how family-focused, community-based interventions can be implemented with refugee families. | Report / case example * | Somali Parents | The article reviews past efforts at delivering family support interventions to refugee families. Application, recommendations for supporting refugee families are provided through a case study. |
| Ponguta et al. 2019, Lebanon | To systematically characterize the implementation and evaluation of MOCEP in Beirut. | A pilot RCT / mixed methods, n = 106 parents | Parents of mixed cultural backgrounds | Despite of the challenges, implementation and evaluations of early childhood parenting programs in fragile contexts are feasible and urgently needed. |
| Puffer et al. 2017, USA | To conduct a RCT assessing the impact of a family-based intervention in Thailand on parenting and family functioning. | RCT, n = 479 families | Burmese migrant families | Both children's and caregivers' reports showed positive treatment effects on parent-child relationship quality across measures. IG caregivers reported significant positive effects of the intervention on overall relationship quality. Positive small effects on the Warmth/Affection subscale for caregivers and children in IG. Greater reductions in negative relationship qualities reported by caregivers and children in IG. Significant, medium-sized effect on reducing harsh punishments in IG. Positive treatment effects on multiple dimensions of family functioning in IG. Most of the improvements in IG remained six months later. |
| Renzaho & Vignjevic 2011, Australia | To evaluate the impact of a culturally appropriate parenting program to reduce intergenerational conflicts and  enhance family cohesion and wellbeing. | Quasi-experimental, single group pretest-posttest, n = 39 families | Sub-Saharan African refugees and migrants | A culturally appropriate parenting skills intervention can assist African migrant families address and overcome some of the difficulties experienced by these parents within the context of an individualistic culture such as Australia. Significantly higher scores (indicating more positive parenting practices) were obtained at follow-up on the dimensions of parental expectations, parental empathy towards children needs, awareness and knowledge of alternatives to corporal punishment, and parent–child family roles. |
| Rivera 2014, USA | To examine the impact of a technology program to assist low-income Spanish-speaking parents in learning and using technology for family advancement. | Quantitative, pretest-posttest surveys, n = 408 parents | Latinx parents | Significant differences were found in the pre- and posttests of parents’ technology skills and their self-efficacy in assisting their children. The program provides technology literacy to parents, creating learning environments for capacity building, as well as empowering parents to become participants in the educational development of their children. |
| Samarasinghe 2011, Sweden | To introduce a socio-politically oriented and community-driven assessment and intervention model. | Theoretical article (introducing a model) | involuntary migrant families in Sweden | The article introduces a conceptual model for assessment and intervention of involuntary migrant families in transition. |
| Sarimski 2013, Germany | To describe the parents' perceptions of the child's possible disability and their attitudes and experiences in relation to early interventions. | Qualitative study, interviews, n = 16 parents, n = 11 childhood education specialists | Turkish migrant parents and childhood education specialists | Early interventions should not generalize the problems to be similar in all migrants. It is important to listen what is important to each family. Access to help (schools, health care) is difficult with language barriers. Culture specific tailoring and intercultural competence is important. |
| Schnur et al. 1995, USA | To describe a program designed to buffer some of the stressors confronting the new immigrant. | Qualitative, interviews, n = 4 mothers | Immigrants from the former Soviet Union | The program helped mothers to cope with the stress, and valued the relationships of their children with the program providers and each other. Mothers emphasized that they appreciated how children had learned English and other academic skills helping them to enter public schools later on. |
| Schulz et al. 2018, Germany | To examine whether more families could be motivated to participate by various financial incentives, and secondly, whether different settings (Triple P individual training vs. -group training) would affect the efficacy. | RCT with follow-up, n = 238 families, and single group study with follow-up, n = 197 families | Migrant and non-migrant families with children aged 2.6 to 6.5 years | Migrants and non-migrants benefited equally from the parental training. Migrants who refused to participate showed significantly less favorable outcomes compared to non-migrants. Regarding the prediction of psychological problems in adolescence, neither MB nor the social status played a role, only psychological problems in early childhood proved to be relevant. |
| Schweitzer et al. 2019, Germany | To describe current practice that combines family-dynamic observations with descriptions of brief family-therapeutic interventions at an outpatient department for psychosocial medicine of an initial registration centre for refugees in Baden-Württemberg. | Qualitative, cases of n = 36 families | Families from nine countries (most of them from Turkey, Syria, Iraq and Iran, Bosnia, Serbia, Kosovo-Albania) in initial registration centre for refugees | Families with a high level of suffering have a need to discuss and a strong desire for quick recovery. Systemic work is often language-based and depending on a good layman interpreters. Narratives of traumatic situations should be listened but clients should not be actively encouraged to tell them. Stabilization and giving resources should be in the foreground. Networking and connections to local social and advice centers, other outpatient clinics, the regional council or leisure groups, is important. The current realities of life of refugee families are more adequate than purely therapeutic ones. |
| Singh et al. 2015, USA | To understand the literacy practices of the refugee families and to understand how the families interacted with the two programs, the Imagination Library free book distribution and ‘‘Storycircles.’’ | Qualitative, ethnography, n = 8 mothers, n = 3 staff members | Burmese and Karen families and library staff members | All had different goals for being a part of the program, ranging from making friends for themselves to creating social spaces for their children, and from learning literacy related concepts themselves to teaching the same to their children. Staff saw difference in the interaction between the parents and the children. Children skills in English increased. Parents started to read books to their children. |
| Slobodin & de Jong 2015, The Netherlands | To examine the evidence for the effectiveness of family interventions  for the range of trauma-related problems among immigrants and refugees. | Systematic literature review, n = 6 studies | Immigrants and refugees (any cultural background) | Based on the review, clear conclusions about the effectiveness of family interventions for traumatized immigrants or refugees cannot be made. Four (n = 4) studies reported results of school-based interventions that included family sessions and two (n = 2) studies described multifamily intervention groups with Kosovar and Bosnian refugees in the United States. |
| Sritharana & Koola 2019, Canada | To understand the barriers that immigrant families of children with ASD face and to describe a culturally sensitive program model to address the barriers. | Literature review, n = 21 studies | Immigrant parents of children with ASD (many cultural backgrounds) | It is important for health care professionals to be aware of different cultural beliefs and work to identify and understand how beliefs regarding child development and ASD may impact the family and child in negative and positive ways. The goals should be to provide culturally sensitive approaches and better access to early interventions. Literature of ASD in migrant children is limited. Evidence for delayed diagnosis of children of ethnic minorities exists. |
| St. Clair & Jackson 2006, USA | To examine the effects of a parent involvement program (MEES) on kindergarten children’s English language skills. | Quasi-experimental, non-randomized controlled trial, n = 29 families | Mostly Hispanic (latinx) families | By the end of first grade, children in IG families scored significantly higher on language measures than children in the CG. |
| St. Clair et al. 2012, USA | To evaluate the long-term effects of the family involvement program on kindergarten children’s English language skills. | Quasi-experimental, non-randomized controlled trial with follow up, n = 50 children | Children from mostly Hispanic (latinx) families | Results demonstrate that children in the IG families scored significantly higher than children in the CG in the follow-up points. |
| Stewart et al. 2015, Canada | To design and evaluate the effects of  an accessible and culturally appropriate social support intervention. | A pilot quasi-experimental study / mixed methods, n = 85 parents | Zimbabwean and Sudanese refugee parents | Increases were found in informational support, spousal support, community engagement, coping, and support-seeking. Decreases were found in parenting stress, loneliness, and isolation. |
| Stewart et al. 2018, Canada | To implement and test an equitable, culturally relevant social support  intervention. | Qualitative, group interviews, n = 67, in-depth individual interviews, n=37 | Sudanese and Zimbabwean refugee parents of young children | The pilot intervention decreased participants’ loneliness and isolation, enhanced coping, improved their capacity to attain education and employment, and increased their parenting competence. Peer mentors who were refugee parents of young children were key to facilitating the support intervention and to enhancing confidence of group members to raise their children in Canada. |
| Umubyeyi & Harris 2012, South Africa | To determine the attitudes and behaviour of refugee mothers concerning the use of corporal punishment and to design a non-violent parenting training course. | Mixed methods, n = 50 mothers | Mothers from Democratic Republic of Congo, Zimbabwe and Burundi | Results:   - changes in mother’s views about corporal punishment - all participants said that they intended to behave differently (e.g. to learn to communicate with their children) |
| Valdez & Martinez 2019, USA | To understand fathers’ experiences with maternal depression in a Mexican immigrant sample. | Qualitative, a dual phenomenological approach, interviews, n = 10 fathers, in-depth case studies, n = 3 fathers | Mexican fathers | Receipt of accurate information about depression increased fathers’ recognition of depression and allowed them to expand traditional gender norms to take an active role in supporting their partners and children. Fathers’ experienced anxiety, shame, loneliness, and helplessness. This study underscores the importance of including fathers in interventions and research on maternal depression in immigrant families. |
| Valdez et al. 2013a, USA | To describe Fortalezas Familiares (FF) a community-based prevention program designed to address relational family processes and promote wellbeing among Latinx families when a mother has depression. | Theoretical article (intervention description) / case study, n = 16 mothers with depressive disorder and their families | Latinx families | The article describes the theoretical basis of the FF program, which is a community-based 12-week intervention for Latinx immigrant women with depression, other caregivers, and their children. |
| Valdez et al. 2013b, USA | To examine the feasibility, acceptability, and preliminary outcomes of an intervention for immigrant Latinx mothers with depression and their families. | A pilot feasibility study / case study, n = 16 families | Latinx mothers with depression and their families | Positive changes following the intervention in psychological functioning, increased family and marital support, and enhanced family functioning, as reported by mothers and other caregivers. Mothers also reported decreased conduct and hyperactivity problems among their children. Children reported positive changes in their psychological functioning and coping, peer relations, parenting warmth and acceptance, and overall family functioning. |
| van den Berg 2016, The Netherlands | To show what constructions of  space-time is used to legitimate parenting training policies? | Qualitative, ethnographic study * | not specified | The article brings together theories of space-time, alterity and ‘cultural lag logics’ in an analysis of a parenting training in the Netherlands. The study shows how societal problems are translated into problems of difference, and how that difference is in turn conceptualized as distance in space and time to be overcome through professional intervention. |
| van Es et al. 2019, The Netherlands | To evaluate the feasibility, acceptability, and potential effectiveness of FAME to reduce parental mental health problems and improve family functioning | Study protocol, mixed-methods approach, target sample size n = 60 families | families living at asylum centres and family locations with children aged 0–18 (culture not specified) | - |
| Warr et al. 2013, Australia | To explore supported playgroup initiative targeting parents and children from non-English speaking backgrounds | Qualitative approach, interviews, n=14 | Parent-facilitators, community worker facilitators and personnel in playgroup coordination | Three key issues emerged: the ways playgroups addressed key barrier for parents in accessing programs (including social isolation and concerns for the risks of involvement with social services), effective community engagement strategies, and the significance of the facilitators’ role in modelling new concepts and parenting approaches. |
| Weine 2011, USA | To describe 8 characteristics that preventive mental health interventions should address to meet the needs of refugee families. | Theoretical article | Refugee families (culture not specified) | The 8 characteristics include: feasibility, acceptability, culturally tailored, multilevel, time focused, prosaicness, effectiveness, and adaptability. |
| Weine et al. 2005, USA | To examine refugee families engaging in the CAFES intervention study | Mixed methods, n = 161 families | Bosnian refugee families | Families that engaged in the intervention experienced more transitions, more traumas, and more difficulties in adjustment. Engagement strategies for multiple-family groups should correspond with the underlying family processes by which refugee families manage transitions, traumas, and adjustment difficulties. |
| Weine et al. 2008, USA | To analyze the effects of a multiple-family group in increasing access to mental health services for refugees with posttraumatic stress disorder (PTSD). | RCT, n = 197 parents | Refugee families from Bosnia-Herzegovina | Multiple-family group was effective in increasing access to mental health services. Depression and family comfort with discussing trauma mediated the intervention effect. |
| Weine et al. 2003, USA | To describe a feasibility study of the Tea and Families Education and Support (TAFES) intervention | Feasibility study (quantitative), n = 86 | Kosovar refugee families | Younger age, fewer children, and higher income were associated with difficulties in engagement to the TAFES intervention. TAFES was able to engage persons who would otherwise not be likely to  seek mental health services. A strength-based family-focused approach used in refugee communities is a feasible approach for engaging a broader range of refugee families into mental health services. |
| Williamson et al. 2014, USA | To pilot the Madres a Madres program. | RCT, n = 194 mother-children dyads | Latinx mothers and their children | Increases in intervention mothers reported parenting skills, family support, and family organization, and reductions in child internalizing behavior from pretest to follow-up, relative to the control condition. |
| Wong et al. 2011, USA | To examine the perceived effectiveness of a brief, community-based parenting  intervention for Vietnamese American immigrant parents. | Qualitative, n = 21 | Vietnamese American immigrant parents | Five themes emerged: (a) increased insight on parent–child relations, (b) need for improvement in communication skills, (c) parent–child cultural gaps, (d) issues of trust between parent and child, and (e) benefits from participation in the workshop. Preliminary evidence for the intervention’s perceived effectiveness was demonstrated by an increase in participants’ intention to show expressive love to and parental empathy for their children. |
| Wong et al. 2013, USA | To examine Mexican origin fathers’ involvement in a family-focused intervention study. | RCT, n = 495 families | Mexican origin families | Higher maternal education and lower economic stress were associated with fathers’ enrollment. Family income was not significantly associated with father enrollment. The interaction between family language and mother report of interparental conflict remained significant in the final model. Probing of the interaction revealed that lower levels of interparental conflict predicted father participation in the study among Spanish-speaking families, but not English-speaking families. Child behavior did not emerge as a significant predictor. |
| Wu & Lee 2015, USA | To address the above-documented challenges facing Asian-American families. | Mixed methods, n = 12 child and parent dyads | Asian-American families | The parents indicated that they gained increased insight into parent-child relationships from participating in the workshop. Parents also reported ambivalence regarding their ability to successfully facilitate their children’s cross-cultural transition. Both parent and child participants reported higher levels of satisfaction within a wide range of psychosocial domains (e.g., personal wellbeing, quality of family relationship, and academic or occupational functioning) upon the conclusion of the workshop. |
| Yagmur et al. 2014, The Netherlands | To test the effectiveness of the VIPP-SD adapted to the specific child-rearing context of Turkish families (VIPP-TM) in the Netherlands | RCT, n = 76 families | Turkish families | Mothers in VIPP-TM intervention showed significant increases in sensitivity and non-intrusiveness whereas mothers in the control group did not show such (strong) improvement. Intervention proved to be effective in increasing maternal sensitivity and non-intrusiveness in these minority families with toddlers at risk for externalizing problems. Maternal discipline strategies were not affected by the intervention. |
| Ying 1999, USA | To test SITICAF’s effectiveness in modifying parenting style, closeness of the intergenerational relationship, and  parent and child’s psychological well-being. | Quantitative, n = 15 parents | Chinese American immigrant parents | Parents reported significant improvement in their sense of efficacy and responsibility for their child’s behavior, and the quality of the intergenerational relationship from pre to post. |
| Ying 2007, USA | To assess the use of SITIF with middle and working class Chinese American immigrant parents. | Quantitative, n = 30 parents | Chinese American immigrant parents | Parents evidenced significant engagement, attending 87% of the classes. Both middle class and working-class parents rated the SITIF as effective in enhancing their parenting and strengthening their intergenerational relationship. |
| Yuen 2019, Hong Kong | To study how target parents evaluates their experiences and the effectiveness of the GPEP. | Qualitative, interviews, n = 4 parents | Low-income and new immigrant parents | Feedback from four parent participants indicated that their knowledge, attitudes, and behaviours regarding parenting empowerment improved. |

* insufficient reporting on research methodology / method section is missing / research methodology unknown

IG = intervention group

CG = control group

WLC = wait-list condition

CPS = Child Protective Services

GPEP = Group parent education programme

USCRI = The U.S. Committee for Refugees and Immigrants

MOCEP = Mother-Child Education Programme

SAFRI = Samarasinghe Refugee Family Intervention Model

ASD = autism spectrum disorder

MEES = Migrant Education Even Start
